# Spatial- and Phospho-Proteomic Profiling Reveals Pancreatic and Hepatic Dysfunction in a Rat Model of Lethal Insulin Overdose

**DOI:** 10.3390/ijms262211018

**Published:** 2025-11-14

**Authors:** Jiaxin Zhang, Shiyi Li, Qian Kong, An He, Mi Ke, Zhonghao Yu, Yuxuan Wang, Xiao Long, Yuhao Yuan, Ruijun Tian, Yiwu Zhou

**Affiliations:** 1 Department of Forensic Medicine, Tongji Medical College, Huazhong University of Science and Technology, Wuhan 430030, China; d202181694@hust.edu.cn (J.Z.); zhonghaoyu795@163.com (Z.Y.); d202381918@hust.edu.cn (Y.Y.); 2 Department of Chemistry, College of Science, Southern University of Science and Technology, Shenzhen 518055, China; lishiyi126@126.com (S.L.); kongq@sustech.edu.cn (Q.K.); hea@sustech.edu.cn (A.H.); kem@sustech.edu.cn (M.K.); 12432162@mail.sustech.edu.cn (Y.W.); lx1989213@outlook.com (X.L.)

**Keywords:** forensic biomarker, insulin overdose, phosphoproteomics, proteomics, spatial proteomics

## Abstract

Insulin, a pivotal hormone synthesized by the pancreas and regulated through hepatic first-pass metabolism, plays an essential role in the management of diabetes. However, non-therapeutic exposure to insulin can lead to life-threatening hypoglycemia. The postmortem diagnosis of fatalities resulting from exogenous insulin presents numerous forensic challenges, including the disruption of pharmacokinetic evidence due to the rapid degradation of insulin after death and the lack of pathognomonic histopathological markers. These factors create significant obstacles in establishing medicolegal causality. Furthermore, the mechanisms underlying insulin overdose-induced injury to the pancreas and liver are poorly understood. This study aims to address these gaps by integrating standardized histopathology, precision laser microdissection, and advanced proteomics to systematically profile the global proteome and phosphoproteome of the liver and pancreas. Furthermore, it includes spatially resolved proteomic mapping of pancreatic microcompartments (islets versus acini) in models of insulin overdose. Comparative analysis with controls revealed dysregulated proteins and phosphorylation sites, along with perturbations in metabolic pathways, primarily affecting pancreatic exocrine and hepatic function. Cross-organ comparative analysis elucidated organ-specific alterations in proteins and phosphorylation sites, uncovering core functional perturbations in these vital organs. In conclusion, this study presents a multi-level proteomic resource that profiles insulin-overdosed rat models and provides insights into the core pathological and molecular signatures.

## 1. Introduction

Insulin, a pivotal peptide hormone secreted by pancreatic β-cells, is essential for regulating glucose homeostasis [1]. Since its isolation and the demonstration of its glucose-lowering effects in 1921, insulin therapy has revolutionized diabetes management, particularly transforming type 1 diabetes mellitus (T1DM) from a fatal disease into a manageable chronic condition [2]. The widespread availability of this life-saving hormone has also facilitated its intentional misuse and abuse [3]. Insulin overdose can cause severe, potentially fatal hypoglycemia. In forensic medicine, the postmortem determination of exogenous insulin as the cause of death remains challenging owing to rapid insulin degradation and nonspecific pathology [4]. Current approaches include detecting residual insulin at injection sites; using high-sensitivity assays to measure abnormally elevated insulin in cardiac blood, vitreous humor, or tissue homogenates in conjunction with a disproportionately low C-peptide level [5,6,7]; and investigating neuropathological auxiliary markers [8]. Nonetheless, systematic studies of such deaths are limited, and the underlying lethal mechanisms remain poorly understood. Therefore, a clearer understanding of the pathological and molecular basis is essential not only for more accurate forensic diagnosis but also for guiding the management of severe hypoglycemia in insulin-treated patients.

The pancreas and liver are pivotal in the regulation of endogenous insulin synthesis, secretion, and metabolic clearance [9]. Histologically, the pancreas comprises exocrine tissue, which facilitates enzymatic digestion, and endocrine islets of Langerhans. The islets, rich in insulin-producing β-cells, regulate glucose homeostasis through the coordinated release of hormones such as insulin and glucagon [10]. Newly synthesized insulin enters the portal circulation and undergoes substantial hepatic first-pass extraction (>50%), which significantly influences systemic bioavailability [9,11]. Notably, dysglycemic states exert profound organ-specific perturbations: fetal hypoglycemia disrupts pancreatic developmental programming, hyperinsulinemic hypoglycemia reflects inherent β-cell secretory dysregulation [12], and iatrogenic hypoglycemia from exogenous insulin administration induces hepatic metabolic dysfunction coupled with aberrant expression of genes such as *GLUT-1* (LC2A1), angiotensinogen (AGT), and *MKP-1* (DUSP1) [13]. Despite the unequivocal centrality of both organs to insulin physiology, the specific responses of pancreas and liver to exogenous insulin overdose—including systematic histological profiling and underlying molecular architecture—remain poorly understood. Addressing this gap could enable the discovery of tissue biomarkers relevant to insulin fatalities and provide mechanistic insight with clinical implications.

In recent years, the rapid advancement of proteomics technologies has significantly propelled research into the mechanisms of liver and pancreatic diseases and their clinical translation. By enabling direct analysis of proteins and their regulatory networks, proteomics has become a crucial tool for investigating both physiological and pathological processes. For instance, Li et al. applied proteomics to delineate dynamic protein expression and stromal-specific changes during human pancreatic development and maturation [14]. Zhang et al. integrated multi-omics data to systematically uncover molecular characteristics and potential therapeutic targets of pancreatic ductal adenocarcinoma [15]. Spatial proteomics techniques, such as region-specific analysis coupled with laser microdissection, have also been applied in pancreatic and liver research [16]. Nevertheless, critical knowledge gaps persist regarding organ-specific proteome dynamics in response to exogenous insulin overdose exposure: systematic global proteome profiling of the liver and pancreas (notably the latter with its marked functional compartmentalization) remains unaddressed; phosphoproteomics data—essential for decoding insulin signaling cascades centered on reversible phosphorylation (e.g., IRS/PI3K/AKT pathways [17,18]) that drive hypoglycemia-related pathological damage—are entirely lacking; and spatial proteome heterogeneity between pancreatic islets and exocrine acini remains uncharacterized due to constraints of conventional tissue homogenization approaches.

To resolve these gaps, this study integrates histopathological assessment, laser capture microdissection (LCM), an integrated spintip-based proteomics technology (Simple and Integrated Spintip-based Proteomics Technology, SISPROT) for the streamlined processing of protein digests from trace tissue samples prior to mass spectrometry, and mass spectrometry-based proteomics to construct a multidimensional proteome atlas in insulin-overdosed Sprague-Dawley (SD) rat models. Our approach encompasses: global proteome profiling of tissue samples (>5000 non-redundant proteins), phosphoproteomics via Ti^4+^-immobilized metal affinity chromatography (Ti^4+^-IMAC) (>6000 high-confidence phosphosites), and spatially resolved proteomic mapping of formalin-fixed pancreatic microstructures (islets and acinar compartments) yielding >3500 identified proteins. Through integrated bioinformatic interrogation, we delineated differential proteome/phosphoproteome signatures across insulin-overdosed versus control tissues (hepatic/pancreatic acinar/islet compartments) and characterized pathology-driven alterations in tissue-enriched proteins (organ-predominant expression) through cross-organ comparative analysis (pancreas vs. liver). This multidimensional dataset establishes an invaluable molecular resource for forensic identification of insulin intoxication fatalities and clinical investigations.

## 2. Results

### 2.1. Overview of the Study and Biological Investigation

**Figure 1:**
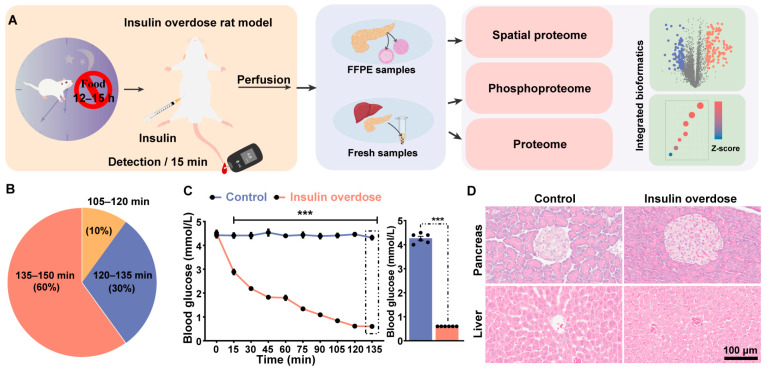
Model construction and proteome profiling of the insulin-overdose rat. (**A**) The workflow schematic outlines the experimental process, starting with establishing the insulin-overdose rat model, followed by the collection and processing of diverse samples into peptides, and execution of mass spectrometry protocols. (**B**) Distribution of time to death in rats following intraperitoneal (i.p.) administration of 20 IU exogenous insulin, n = 10. (**C**) Temporal changes in blood glucose of insulin-overdosed rats were analyzed by two-way ANOVA followed by Sidak’s multiple comparisons test. Data are presented as the mean ± SEM. *** *p* < 0.0001 vs. control group at respective time points. The blue bar represents the control group, while the red bar represents the insulin-overdose group. (**D**) Histopathological features of liver and pancreas in control vs. insulin-overdose groups (HE staining). (Upper Left) Control pancreas exhibits intact architecture with uniform eosinophilic staining in acinar cells; (Upper right) Insulin-overdose pancreas shows reduced eosinophilia, cellular edema in acinar cells, and focal edema in islets; (Bottom left) Control liver displays normal hepatocyte morphology; (Bottom right) Insulin-overdose liver demonstrates cellular edema and congestion.

An acute exogenous insulin poisoning model was established in SD rats to systematically profile the proteomic alterations induced by insulin overdose. Pancreatic tissues were perfusion-fixed with 4% paraformaldehyde (PFA), embedded in paraffin, and precisely dissected using laser microdissection technology to isolate islet and acinar regions for spatially resolved proteomic analysis. Simultaneously, fresh liver and pancreatic tissue samples were rapidly collected for global proteomics and phosphoproteomics analyses, respectively, to capture molecular events triggered by insulin overdose (Figure 1A).

An acute hypoglycemic model was established by intraperitoneal injection of a lethal insulin dose (20 IU/kg). Among the ten treated rats, the time to death ranged from 1.75 to 2.5 h post-injection, with mortalities distributed as follows: 1 (10%) at 1.75–2 h, 3 (30%) at 2–2.25 h, and 6 (60%) at 2.25–2.5 h (Figure 1B). To monitor blood glucose dynamics in real-time, tail vein blood sampling was performed every 15 min post-injection (Figure 1C). Rats in the insulin overdose group exhibited a significant progressive decline in blood glucose levels over time, accompanied by typical neuroglycopenic symptoms: initial manifestations included reduced activity and impaired responsiveness; when blood glucose dropped to approximately 1.0 mmol/L, irregular limb convulsions occurred; later progressing to severe neurological excitation symptoms such as opisthotonosis, indicating a moribund state. Ultimately, all rats succumbed. Blood glucose levels dropped below the detection limit of the glucometer (0.6 mmol/L) by 120 min post-injection; subsequent readings were recorded as 0.6 mmol/L. In contrast, blood glucose remained stable in the control group throughout the observation period.

Acute liver injury is a recognized consequence of excessive insulin administration in clinical reports [19]. Previous research has also shown that lethal insulin overdose in rats results in hepatic damage, primarily characterized by congestion in the central hepatic veins and hepatic sinusoids [20,21], which is consistent with our findings. Hematoxylin and Eosin (HE) staining revealed mild hepatic edema and hepatic congestion. Furthermore, acute hypoglycemia has been linked to reduced insulin secretion [22], suggesting a potential impairment of pancreatic function under such conditions. To investigate the potential histological basis of this phenomenon, we performed HE staining on pancreatic tissue. The results showed mild edema in some pancreatic islet cells, along with cellular edema and reduced eosinophilia in the acinar structures. These structural changes may be associated with the aforementioned functional suppression.

### 2.2. Spatial Proteome Profiling Reveals the Dysregulation of the Digestive Zymogens in Acinus and Disruption of Proteostasis in Islet

**Figure 2:**
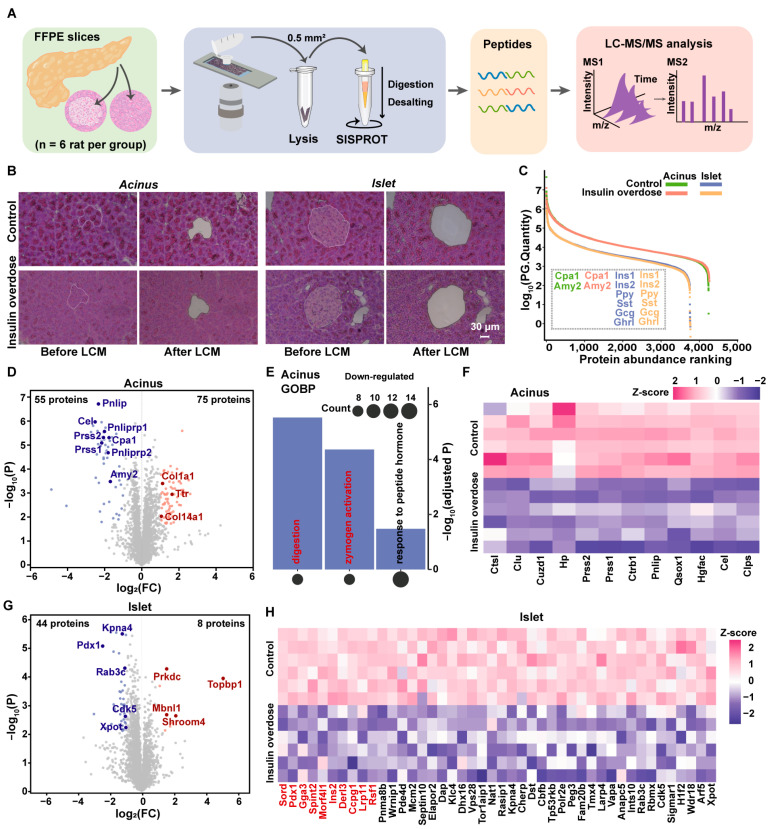
Spatial proteomic signatures of distinct pancreatic regions in a rat model of insulin overdose. (**A**) Spatial proteomics workflow for FFPE pancreas islet and acinus. (**B**) Representative HE staining demonstrating LCM of pancreatic acinus and islet from control and insulin-overdose groups for spatial proteome profiling. Scale bar = 30 μm. (**C**) Dynamic range of the pancreatic proteome in acinar and islet regions from control and insulin-overdose groups. Key cell-type-specific marker proteins are labeled. Curves are color-coded: control acinus (green), control islet (blue), insulin-overdose acinus (red), insulin-overdose islet (orange). n = 6 per group. (**D**,**G**) Volcano plots comparing proteomic alterations in insulin-overdose versus control pancreatic tissues. Acinus (**D**) region showing upregulated (red) and downregulated (blue) proteins; (**G**): Islet region. Differentially expressed proteins satisfying |Log_2_FC| ≥ 1 with adjusted *p* < 0.05. Representative proteins labeled at corresponding images and positions. (**E**) The bar graph showing the GOBP terms for the downregulated proteins in the insulin-overdose group. The size of the circle represents the number of proteins, and the *Y*-axis indicates the −log_10_(adjusted P). (**F**–**H**) Heatmaps showing Z-score normalized quantities of downregulated proteins in the acinus ((**F**), showing proteins involved in digestion and zymogen activation) and islet ((**H**), highlighting the top 10 proteins with the highest fold change) of insulin-overdose versus control groups. The color scale represents Z-scores, with red indicating high expression and blue indicating low expression.

To investigate region-specific proteomic alterations in pancreatic tissues, LCM was employed to isolate acinar and islet regions from control and insulin-overdosed rats (~0.05 mm^2^ per sample; Table S1). Microdissected formalin-fixed paraffin-embedded (FFPE) specimens were processed using the SISPROT technique [23,24,25], which is optimized for minimal sample inputs, followed by liquid chromatography-tandem mass spectrometry (LC-MS/MS) analysis (Figure 2A,B). A total of 4265 acinar and 3777 islet proteins were quantified (Control group: 4243 acinar/3767 islet; Insulin-overdosed group: 4261 acinar/3768 islet). High intra-group reproducibility was observed (Figure S1A). Principal component analysis (PCA) revealed clear separation between groups in both tissue types, confirming the model’s successful establishment (Figure S1B). To validate cell-type specificity and microdissection accuracy, enriched expression of islet marker proteins—Ins1/In2 (β-cells), Gcg (α-cells), Sst (δ-cells), Ppy (γ-cells), and Ghrl (ε-cells)—was detected in islet regions, while Cpa1 (acinar cells) was highly expressed in acinar regions (Figure 2C). These findings confirm the precision of the microdissection and the overall reliability of our proteomic workflow.

To characterize insulin-overdosed proteomic changes and functional pathway dysregulation, differentially expressed proteins (DEPs) in both acinar and islet tissues were identified using two-sample Student’s *t*-tests (FDR < 0.5). Compared to controls, insulin-overdosed acini exhibited 55 downregulated and 75 upregulated proteins, while islets showed 44 downregulated and 8 upregulated proteins (Figure 2D,G). Pancreatic acini, the functional units of the exocrine compartment, synthesize and store zymogens such as alpha-amylase (Amy2), serine protease 1 (Prss1), and anionic trypsin-2 (Prss2) [26,27]. These proenzymes are secreted via ducts to the duodenum, where activation enables nutrient breakdown for intestinal absorption [28]. In acute pancreatitis (AP), acinar digestive enzyme dysregulation correlates histologically with reduced cytoplasmic eosinophilia [29]. Insulin-overdosed models exhibited identical AP-like pathologies (Figure 1D and Figure 2B), with proteomics confirming downregulation of key enzymes (e.g., Amy2; Figure 2D), evidencing disrupted enzymatic homeostasis. Following differential protein screening, stratified Gene Ontology (GO) Biological Process (BP) enrichment analysis of upregulated (Figure S2A) and downregulated (Figure 2E) acinar proteins was performed using Metascape. GOBP revealed downregulated proteins enriched in zymogen activation (Figure 2E). Subsequently, proteins belonging to the top two significantly enriched GOBP terms were selected for visualization of their expression levels via heatmap (Figure 2F). Strikingly, CSTL, which physiologically prevents AP by degrading trypsin(ogen) [30,31], was the most significantly downregulated protein in our dataset (Figure 2F). Analysis of upregulated proteins revealed enrichment in acute injury responses, most significantly “response to wounding” and “acute-phase response” (Figure S2A). DEPs associated with these pathways were visualized via heatmap (Figure S2B), showing prominent enrichment of classical markers α1-antitrypsin (Serpina1) and C-reactive protein (CRP) [32,33]-which exhibit characteristically elevated expression in pathologies including acute pancreatitis [33].

The pancreatic islets constitute the endocrine compartment of the pancreas and are responsible for systemic glucose regulation through the secretion of insulin and glucagon [10]. Proteomic analysis revealed the downregulation of key regulators essential for islet development and functional maintenance. Most strikingly, this included the β-cell transcription factor Pdx1 (Figure 2G), a master regulator whose deficiency is known to critically impair β-cell function [34]. Pdx1 was one of the most significantly downregulated proteins (Figure 2H). Its coordinated downregulation with Ins2 points to a extensive disruption of the insulin synthesis pathway. Furthermore, we observed significant downregulation of other critical regulators of β-cell homeostasis, such as Gga3 [35] and Derl3 [36]. Differential upregulation analysis identified only 8 significantly expressed proteins, visualized via heatmap in Figure S2C, including Mbnl1—a protein previously established to regulate pancreatic β-cell function.

### 2.3. Global Proteome Profiling of Insulin-Overdose Rat Models Reveals Metabolic Disorder and the Acute Phase Response

**Figure 3:**
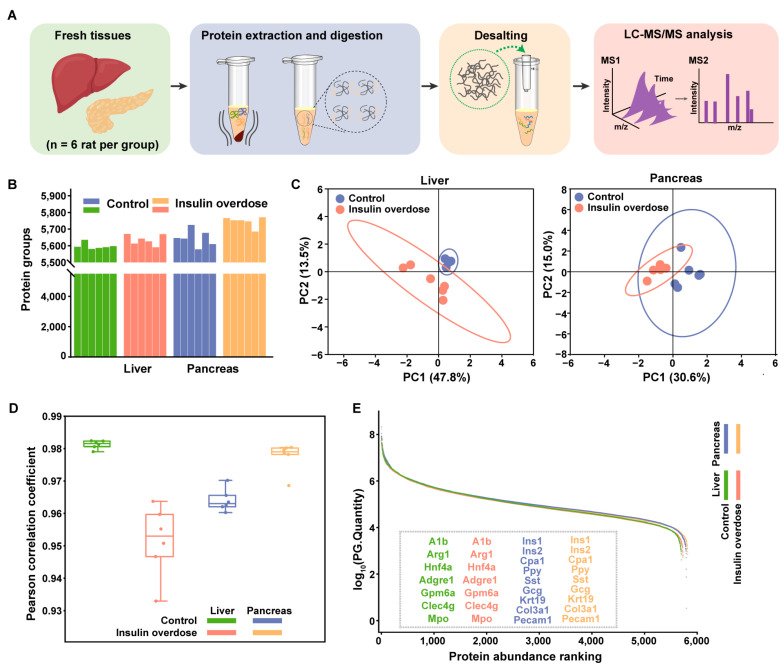
Proteomic data quality assessment in a rat model of insulin overdose: liver and pancreas. (**A**) Proteomics workflow for fresh liver and pancreas tissues. (**B**) Number of protein groups identified in liver and pancreatic tissues under control and insulin-overdose conditions. (**C**) PCA of the liver and pancreas proteomes from control and insulin-overdose rats, n = 6 per group. (**D**) Box-whisker plot of Pearson correlation coefficient in different liver and pancreas groups, respectively. n = 6 per group. (**E**) Dynamic range of liver and pancreas proteome under control and insulin-overdose groups based on average protein PG Quantity. Identified marker proteins for major cell types in the liver or pancreas are annotated in the images, n = 6 per group.

To profile the acute proteomic response to insulin overdose, proteins from freshly harvested rat liver and pancreas were digested in solution and analyzed by LC-MS/MS, as outlined in Figure 3A. Global proteomic profiling identified 5736 proteins in the liver and 5821 proteins in the pancreas (Figure 3B). PCA revealed clear separation between the insulin-overdosed and control groups in both the liver and pancreas (Figure 3C). High intra-group reproducibility was also demonstrated (Figure 3D). The datasets robustly detected key cell-specific markers for major cellular populations [37]. In pancreatic tissue, this included markers for acinar cells (CPA1), β-cells (Ins1/2), α-cells (Gcg), and δ-cells (Sst), ductal cells (Krt19), Stromal cells (Col3a1), and endothelial cells (Pecam1). Similarly, hepatic markers for hepatocytes (Alb, Hnf4α, Arg1), sinusoidal endothelial cells (Clec4g), mesothelial cells (Gpm6a), and Kupffer cells (Mpo) were identified (Figure 3E). These results confirm the extensive coverage of our global proteomic dataset and the robustness of our analytical workflow.

**Figure 4:**
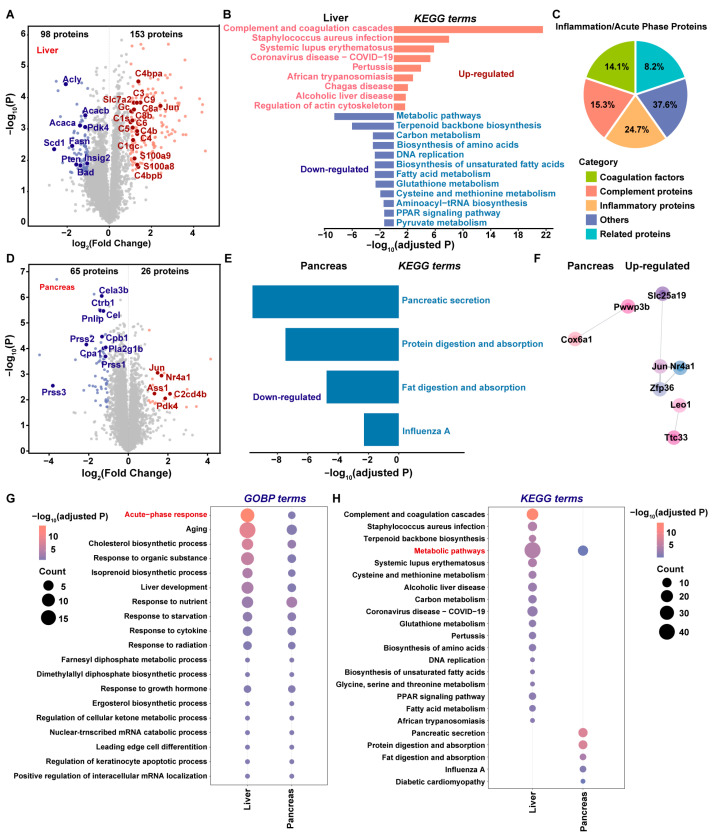
Proteomic-based functional analysis of DEPs in the liver or pancreas. (**A**,**D**) Volcano plots comparing proteomic alterations in insulin-overdose versus control samples. (**A**) Liver showing upregulated (red) and downregulated (blue) proteins; (**D**) pancreas. DEPs satisfying |Log_2_FC| ≥ 1 with adjusted *p* < 0.05. Representative proteins labeled at corresponding images and positions. (**B**,**E**) KEGG enrichment analysis of DEPs in insulin-overdose liver and pancreas tissues. The bar lengths indicated the adjusted *p*-value magnitude during enrichment analysis. (**C**) Composition analysis of Inflammatory/Acute-Phase Proteins among DEPs in the livers of rats with insulin overdose. (**F**) PPI network analysis of up-regulated proteins in the pancreas under insulin overdose conditions. (**G**,**H**) Dot plot of significant GOBP (**G**) and KEGG (**H**) terms for DEPs in the liver and pancreas. Dot color indicates the adjusted *p*-value significance, and size indicates the number of associated proteins. Pathways labeled in red text represent key targets.

Based on the clear separation observed in the PCA, DEPs and pathway enrichments were analyzed to delineate the response to insulin overdose. In the liver dataset, the insulin-overdosed group showed 153 upregulated and 98 downregulated proteins (Figure 4A). Kyoto Encyclopedia of Genes and Genomes (KEGG) enrichment analysis identified the complement and coagulation cascades as the most significantly enriched pathways, indicating that insulin overdose triggered an inflammatory response and acute phase reaction (Figure 4B). This was further supported by the substantial upregulation of complement components, coagulation factors, inflammatory mediators, and related proteins (Figure 4C). Given that the liver is the primary site for the synthesis of these factors, these findings suggest that it may serve as a central amplifier of immune and inflammatory signaling during insulin-induced lethality. In contrast, the downregulated proteins were predominantly associated with energy metabolism, including key hepatic lipogenic regulators such as Acaca, Acacb, Fasn, Scd1, and Acly. Pathway enrichment analysis confirmed the pervasive suppression of metabolic processes (Figure 4B), indicating a marked compromise in hepatic metabolic function.

Global proteomic analysis of the intact pancreas identified 26 upregulated and 65 downregulated proteins in the insulin-overdosed group compared to controls (Figure 4D). Among these, the dataset revealed significant downregulation of key digestive enzymes (Prss1, Prss2, Prss3, and Cpa1). This finding was consistent with spatial proteomics data, together providing strong evidence for an insulin-overdose-induced impairment of pancreatic exocrine function. Pathway enrichment analysis further revealed that the downregulated proteins were predominantly associated with pancreatic secretion, protein digestion and absorption, and fat digestion and absorption, underscoring disruptions in pancreatic exocrine function (Figure 4E).

As the upregulated pancreatic proteins showed no significant enrichment in KEGG pathways, a protein-protein interaction (PPI) network analysis was conducted to explore their functional relationships (Figure 4F). The results revealed a relatively limited number of interactions among the upregulated proteins. Among these, interactions between Jun, Nr4a1, and Zfp36 have been reported in multiple studies [38,39]. These proteins are primarily involved in inflammation [40] and stress response, and are classified as immediate-early gene products. Their upregulation suggests a stress response in the pancreas under conditions of insulin excess, potentially accompanied by inflammatory processes. Furthermore, Nr4a1 (mainly expressed in pancreatic β-cells) exhibits glucose concentration-dependent regulation and modulates insulin secretion [41]. Together, these findings indicate that insulin excess may lead to pancreatic damage, impairing both its exocrine and endocrine core physiological functions.

To further investigate the functional connection between the liver and the pancreas under insulin overdose conditions, we performed comparative GOBP and KEGG pathway analyses on all DEPs identified in the liver and pancreas, respectively. GOBP analysis demonstrated that both the liver and pancreas underwent acute responses, with notably stronger expression observed in the liver (Figure 4G). In contrast, KEGG enrichment analysis revealed that the liver and pancreas overlapped only in metabolic pathways, suggesting that insulin overdose induced systemic metabolic alterations in both organs (Figure 4H).

In summary, global proteomic profiling in a rat model of exogenous insulin overdose demonstrated a pronounced acute and inflammatory response in the liver, concomitant with impaired pancreatic secretory function and widespread disruption of systemic metabolic pathways in both organs.

### 2.4. Phosphoproteome Profiling of Insulin-Overdose Rat Models Reveals Metabolic Pathway Dysregulation

**Figure 5:**
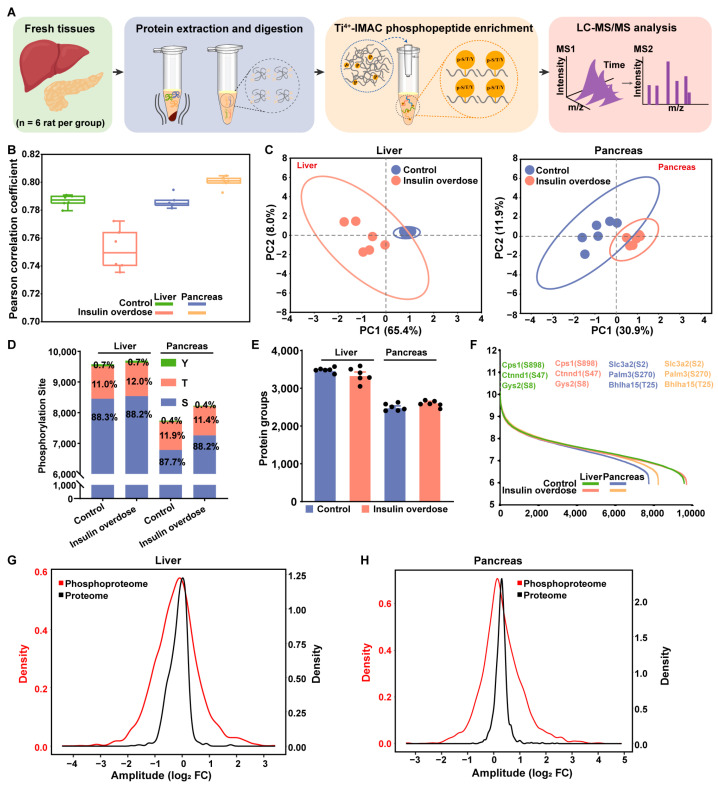
Phosphoproteomic data quality assessment in a rat model of insulin overdose: liver and pancreas. (**A**) Phosphoproteomics workflow for fresh liver and pancreas tissues. (**B**) Box-whisker plot of Pearson correlation coefficient in different liver and pancreas groups, respectively. n = 6 per group. (**C**) PCA of phosphoproteomics profiles in the liver and pancreas from Insulin overdose rats and Control rats. (**D**) Quantification of phosphoproteins in liver and pancreatic tissues under control and insulin overdose conditions. (**E**) Bar plot showing analysis of phosphorylation sites in liver and pancreas tissues under control and insulin overdose conditions. Bar colors represent three phosphorylation types: green (Y, tyrosine), blue (S, serine), and red (T, threonine). (**F**) Dynamic range of liver and pancreas phosphoproteome under control and insulin overdose groups based on average protein PG.Quantity. The confirmed liver- and pancreas-specific phosphorylation sites are presented in the image. n = 6 per group. (**G**,**H**) Density plots comparing the phosphopeptides and the corresponding proteins of liver (**G**) and pancreas (**H**), respectively.

Based on reports of hypoglycemia-mediated organ regulation via protein phosphorylation [42], this study characterized hepatic and pancreatic phospho-signaling dynamics in response to insulin overdose. Phosphoproteomic analysis followed the workflow in Figure 5A, with sample pretreatment aligned with global proteome processing. Phosphopeptides were enriched via Ti^4+^-IMAC [43], followed by LC-MS/MS analysis. In these datasets, high intra-group reproducibility was demonstrated (Figure 5B), and PCA revealed clear separation between the insulin-overdosed and control groups in both liver and pancreas (Figure 5C). Phosphoproteomic quantification identified approximately 9352 phosphosites (4075 phosphoproteins) and 7643 phosphosites (3223 phosphoproteins) in control liver and pancreatic tissues, respectively; these increased to 9456 (4123 proteins) and 8137 (3341 proteins) in insulin-overdosed tissues. All datasets included S/T/Y typing (Figure 5D,E). Furthermore, this study ranked the identified proteins in each group of samples based on their expression levels and detected multiple known phosphorylation sites, including Cpa1 (S898), Ctnnd1 (S47), and Gys2 (S8) in the pancreas, as well as Slc3a2 (S2), Palm3 (S270), and Bhlha15 (T25) in the liver (Figure 5F). To evaluate the dependency of phosphorylation dynamics on protein abundance, quantitative comparison of phosphopeptide levels and corresponding protein abundances revealed that phosphorylation changes significantly exceeded alterations in protein abundance (Figure 5G,H), indicating protein abundance-independent regulatory functions under insulin overdose.

To identify phosphorylation sites independent of proteomic fluctuations, a dual screening strategy was implemented: (1) Primary screening required |Log_2_FC (phosphoproteomics)| > 1 and |Log_2_FC (phosphoproteomics)| > |Log_2_FC (proteomics)| (Figure S3A); (2) The secondary screening step required |Log_2_FC (proteomics)| > 1 and FDR < 0.05, based solely on phosphoproteomic data analysis. The intersection of the two screening criteria was visualized using a Venn diagram, which represents the final set of significantly altered phosphosites identified (Figure S3B). This yielded 508 phosphorylation sites in liver (338 upregulated/170 downregulated) and 545 in pancreas (447 upregulated/98 downregulated), representing post-translational regulatory events decoupled from proteomic alterations.

To investigate the functions of the differentially phosphorylated sites, we performed KEGG pathway enrichment analysis on the corresponding proteins.

**Figure 6:**
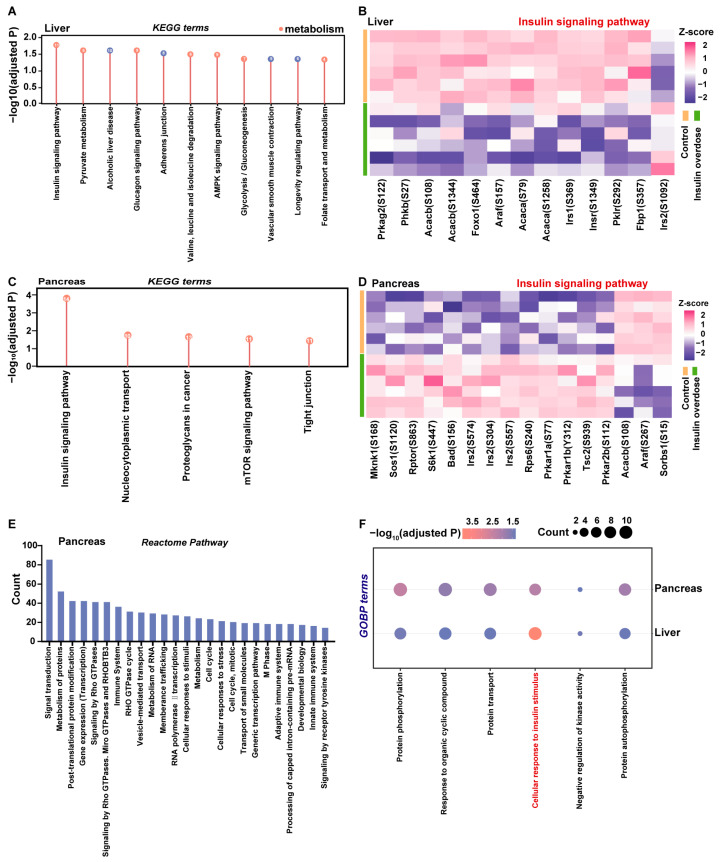
Phosphoproteomic-based functional analysis of DEPs of the liver or pancreas. (**A**,**C**) KEGG enrichment analysis of DEPs in insulin-overdose liver and pancreas tissues. The bar lengths indicate the adjusted *p*-value during enrichment analysis. (**A**): The color coding of the dots is used to categorize pathways functionally: red dots represent metabolism-related pathways, while blue dots represent non-metabolism-related pathways. (**B**,**D**) Heatmap showing altered phosphorylation sites on insulin signaling pathway-related proteins in the liver (**B**) and pancreas (**D**) samples. Protein expression levels were Z-score. (**E**) Reactome pathway enrichment analysis of differentially phosphorylated proteins induced by insulin overdose in pancreatic tissue. The height of the bars corresponds to the number of differentially phosphorylated proteins enriched in respective pathways. (**F**) Dot plot displaying the significant GOBP terms for differentially phosphorylated proteins obtained in the liver and pancreas. The color gradient of the dots represents the adjusted *p*-value magnitude, while the dot size corresponds to the number of proteins involved in the term.

In the liver, the significantly enriched pathways were predominantly metabolism, including the insulin and the AMPK signaling pathway, indicating that the liver may dynamically regulate energy metabolism through phosphorylation in response to insulin overdose (Figure 6A). A heatmap was generated to visualize the overall phosphorylation changes in enriched proteins and their specific sites within the insulin signaling pathway (Figure 6B). Notably, the functions of several sites, including Insr (S1349), Irs1 (S369), and Irs2 (S1092), remain unclear. However, multiple known sites on Irs1 or Irs2 (e.g., S307 and S318 of IRS1) have been demonstrated to regulate the insulin signaling pathway [44,45], and studies indicate that phosphorylation responses at distinct Irs sites are stimulus- and time-dependent [46,47]. Therefore, the phosphorylation changes observed at Irs1 (S369) and Irs2 (S1092) in this study suggest their potential involvement in modulating the insulin signaling pathway under conditions of insulin overdose.

In the pancreas (Figure 6C), the insulin and mTOR signaling pathways were significantly enriched, reflecting the impact of insulin overdose on pancreatic metabolism. Given the notable enrichment of the insulin signaling pathway, we further examined the phosphorylation states of key proteins. As shown in Figure 6D, phosphorylation levels at S304, S557, and S574 of Irs2, a key downstream protein of the insulin receptor, were significantly upregulated, though the functions of these sites have not been clearly defined. Sequence alignment revealed that rat Irs2 S304 and S574 correspond to mouse Irs2 S303 and S573 [48], respectively. Since the latter have been confirmed to regulate insulin signaling, it is plausible that the corresponding sites in rats may serve similar functions. Concurrently, enhanced phosphorylation of Rptor at S863, a key component of mTORC1, along with increased phosphorylation of downstream S6k1 at S447 and its target RPS6 at S240, indicated activation of the Irs2–mTORC1–S6k1 signaling axis. Thus, these results not only confirm phosphorylation changes at known key sites but also identify novel phosphorylation sites on Irs2 and S6k1, providing new molecular insights into how insulin excess affects pancreatic function. Subsequent Reactome pathway analysis (Figure 6E) further demonstrated that the differentially phosphorylated proteins were enriched in processes such as signal transduction and protein metabolism, consistent with the KEGG findings.

While KEGG analysis revealed shared enrichment in the insulin signaling pathway, tissue-specific responses diverged: pancreatic pathways were primarily associated with cell proliferation and growth, whereas hepatic responses centered on metabolism and energy regulation. To further compare the similarities and differences in phosphorylation-mediated responses between the liver and pancreas under insulin overdose, we performed GOBP analysis (Figure 6F). Both tissues showed significant enrichment in “protein phosphorylation” and “cellular response to insulin stimulus,” indicating that phosphorylation is a common mechanism in both organs in response to insulin overdose, and both exhibit clear insulin-induced physiological and molecular adaptations.

In summary, this study systematically revealed the signaling regulation in the liver and pancreas under insulin overdose through phosphoproteomic analysis and identified several uncharacterized phosphorylation sites on key proteins, providing new clues and research directions for in-depth dissection of the precise regulatory mechanisms of the insulin signaling pathway.

## 3. Discussion

This study established a rat model of lethal insulin overdose and applied an integrated approach combining global proteomics, spatially resolved proteomics, and phosphoproteomics to systematically characterize molecular alterations in the liver and pancreas under insulin overdose conditions.

In the pancreas, insulin overdose caused marked downregulation of key digestive enzymes (such as amylase), indicating impaired exocrine function. Conversely, the liver exhibited suppressed metabolic pathways alongside upregulated expression of inflammatory and complement proteins, suggesting concomitant metabolic dysfunction and inflammatory activation. Common alterations in both organs were primarily observed in the acute phase response and metabolic pathway disruptions, indicating that insulin overdose-induced organ injury shares conserved features while also exhibiting tissue-specific responses.

Furthermore, phosphoproteomic analysis yielded deeper mechanistic insights, revealing not only extensive dysregulation of phosphorylation within insulin signaling and metabolic pathways but also a significant enrichment of the GOBP terms “cellular response to insulin stimulus”, which collectively underscore the centrality of insulin signaling disruption. Moreover, this study identified multiple significantly altered yet functionally uncharacterized phosphorylation sites, offering new clues for understanding the effects of insulin overdose.

It is noteworthy that the differential molecular alterations identified in this study demonstrate significant translational potential in forensic medicine. Current postmortem diagnosis of fatal insulin overdose lacks specific biological biomarkers. The specific molecular changes identified in the pancreas and liver offer promising avenues for developing novel diagnostic approaches. Future analysis of the expression levels of these markers in postmortem pancreatic and hepatic tissues could provide objective laboratory evidence for determining fatal insulin overdose in complex cases. Nevertheless, this prospective application requires further validation in human samples.

We emphasize that the primary contribution of this work is its discovery-phase nature. We have revealed a comprehensive molecular landscape and identified potential key targets and pathways, thereby establishing a solid foundation for subsequent in-depth mechanistic investigations. While the current findings are primarily descriptive, they provide valuable insights into the pathological alterations and underlying molecular mechanisms in the liver and pancreas under lethal insulin overdose conditions, while also laying an important foundation for future research.

### Limitations of the Study

First, the study relied on an SD rat model with a modest sample size and lacked validation using human clinical samples. Second, the research strategy focused on data acquisition and descriptive analysis at the levels of global proteome, phosphoproteome, and spatially resolved proteomics of the pancreas (specifically targeting islet and acinar structures), with no extension to in-depth causal or functional mechanistic investigations. Third, the omics analyses captured molecular states at a single terminal time point and thus could not reveal the temporal dynamics of key proteins or modifications during disease progression.

## 4. Materials and Methods

### 4.1. Animals

Male SD rats (7–8 weeks) were purchased from the Laboratory Animal Center of Huazhong University of Science and Technology. Rats were housed under controlled environmental conditions at 25 °C with a 12-h light/dark cycle and provided ad libitum access to autoclaved food and sterile water. All animal experiments received approval from the Institutional Animal Care and Use Committee (IACUC) of Huazhong University of Science and Technology (Approval No. 4674, 2024).

### 4.2. Rat Model of Exogenous Insulin-Induced Overdose

We established the animal model as previously described [20]. Prior to experiments, all rats were fasted for 12–15 h with free access to water. Phase I: To determine the mortality time interval, rats were intraperitoneally administered exogenous insulin (Recombinant human insulin injection, Novo Nordisk, Copenhagen, Denmark) at 20 IU/kg, with blood glucose levels assessed via tail vein sampling using a clinical-grade glucometer (Accu Chek Guide blood glucose meter, Roche, Mannheim, Germany) at 15-min intervals until biological death confirmation. Phase II: A total of 24 rats were randomly assigned into two groups: the control group and the insulin overdose group. In the insulin overdose group, model establishment followed the aforementioned protocol. At 135 min post-insulin administration, rats were anesthetized using a small animal ventilator (RWD Life Science, Shenzhen, China) delivering 3–4% isoflurane. Subsequently, transcardial perfusion was performed, followed by systematic dissection of the liver and pancreas.

During perfusion, each group was divided into two cohorts: Subgroup A underwent transcardial perfusion with ice-cold phosphate-buffered saline (PBS) for rapid harvest of fresh liver/pancreas, immediately snap-frozen in liquid nitrogen-cooled isopentane (−80 °C) for proteomic and phosphoproteomic analyses. Subgroup B received PBS perfusion followed by 4% PFA fixation for paraffin-embedding and spatial proteomics. Control groups received intraperitoneal saline instead of insulin, with all other procedures identical to the insulin-overdosed group.

### 4.3. HE Staining

Hepatic and pancreatic specimens were fixed, ethanol-dehydrated, and paraffin-embedded for room-temperature storage. Sections (4 μm) were cut using a rotary microtome and mounted on adhesive slides. Standard HE staining was performed [23], followed by whole-slide digital scanning. All sections underwent blinded evaluation by ≥2 pathologists.

### 4.4. Laser Microdissection

Poly-L-lysine-coated membrane slides (optimized for laser microdissection) replaced conventional slides for 4 μm tissue sections. After H&E staining and digital scanning, targeted pancreatic acini and islets with pathological alterations were isolated using an MMI CellCut system [49]. Gross localization (4× magnification) preceded precision microdissection (40×). Specimens reaching 0.5 mm^2^ collection area were transferred to polymerase chain reaction (PCR) tubes for downstream analysis.

### 4.5. Slice Sample Processing for Protein Extraction and Digestion

Microdissected specimens were pretreated using the SISPROT SE Kit (BayOmics, Shenzhen, China). Tissue slices in PCR tubes with 30 μL lysis buffer underwent 30 cycles of non-contact sonication [30 s ON/OFF, 4 °C; Bioruptor (Diagenode, Liège, Belgium)], followed by thermal denaturation at 90 °C for 90 min and a subsequent 30-min sonication at 4 °C. The kit-provided sample processor was centrifugally activated, followed by enzymatic digestion at 37 °C for 1 h, and peptide elution for LC-MS/MS analysis.

### 4.6. Fresh Sample Processing for Protein Extraction and Digestion

Liver and pancreatic tissues were lysed in ice-cold buffer [50 mM Tris pH 8.5, 7 M urea (Sigma-Aldrich, St. Louis, MO, USA), 1% Triton X-100 (Sigma-Aldrich, St. Louis, MO, USA), 10 nM sodium fluoride [NaF (Sangon Biotech, Shanghai, China)], 1 mM sodium orthovanadate [Na_3_VO_4_ (Sangon Biotech, Shanghai, China)], 1× protease/phosphatase inhibitors (Roche, Basel, Switzerland)] using a tissue grinder, followed by 40-min ice incubation. Protein-containing supernatant was quantified by bicinchoninic acid [BCA (Thermo Fisher Scientific, Waltham, MA, USA)] assay. For each 2 mg protein aliquot in a 15 mL tube, sequential addition of methanol (Sigma-Aldrich, St. Louis, MO, USA)/chloroform (Sigma-Aldrich, St. Louis, MO, USA)/ice-water enabled phase separation (4000× *g*, 15 min). Interphase proteins were methanol-resedimented (4000× *g*, 5 min), then dissolved in 8 M urea buffer (200 μL/mg protein) via sonication. Proteins underwent disulfide reduction [10 mM dithiothreitol (DTT, Sigma-Aldrich, St. Louis, MO, USA), 55 °C, 30 min], alkylation [30 mM iodoacetamide (IAA, Sigma-Aldrich, St. Louis, MO, USA), room temperature (RT), 20 min dark], and quenching (20 mM DTT). After urea dilution to <2 M with 50 mM Tris pH 8.5, trypsin (Sigma-Aldrich, St. Louis, MO, USA) digestion [1:20 *w*/*w* enzyme/protein ratio, 1 mM calcium chloride (CaCl_2_, Sigma-Aldrich, St. Louis, MO, USA)] proceeded at 37 °C for 16–18 h. Acidification with trifluoroacetic acid (TFA, Thermo Fisher Scientific, Waltham, MA, USA) (pH 2–3) terminated digestion, followed by C18 desalting [Waters Sep-Pak^®^ (Waters, Milford, MA, USA)]. Following desalting, the peptide sample was aliquoted and lyophilized to dryness. The resulting peptides were then used for phosphopeptide enrichment and global proteome analysis, respectively.

### 4.7. Phosphopeptide Enrichment

Lyophilized peptides were reconstituted in loading buffer [80% acetonitrile (ACN, Thermo Fisher Scientific, Waltham, MA, USA), 6% TFA] for Ti^4+^-IMAC phosphopeptide enrichment [50]. A C8 membrane-packed 200 μL pipette tip was loaded with Ti^4+^-IMAC beads (20:1 *w*/*w* bead-to-peptide mass ratio), centrifuged to immobilize beads, then loaded with peptides. Sequential washes with Buffer 1 [50% ACN, 6% TFA, 200 mM sodium chloride (NaCl, Aladdin^®^, Shanghai, China)] and Buffer 2 (30% ACN, 0.1% TFA) removed non-specific bindings. Phosphorylated fractions were eluted with 10% ammonium hydroxide (NH_4_OH, Sigma-Aldrich, St. Louis, MO, USA)/50% ACN, lyophilized, and redissolved in 1% formic acid (FA, Sigma-Aldrich, St. Louis, MO, USA). Peptide samples were reconstituted and then desalted using C18 membranes that had been activated with methanol and equilibrated with FA. The target peptides were eluted and collected with a solution of 50% acetonitrile containing 0.5% acetic acid. Finally, the collected elution was lyophilized and stored at −80 °C for LC-MS/MS analysis.

### 4.8. LC-MS/MS Analysis

An Orbitrap Exploris 480 mass spectrometer equipped with a Dionex UltiMate 3000 RSLCnano system (Thermo Fisher Scientific, Waltham, MA, USA) was utilized for LC-MS/MS analysis.

Peptides corresponding to a 0.5 mm^2^ tissue area (Spatial Proteomics) and ~1 μg peptides (Global Proteomics) were separated on a home-made C18 column (20 cm × 100 μm i.d) packed with 1.9 μm C18 beads (120 Å pore size; Dr Maisch GmbH, Tübingen, Germany). Peptide separation was performed using a 90-min gradient. All MS spectra were acquired in data-independent acquisition (DIA) mode, which systematically fragments all ions within sequential mass isolation windows to provide comprehensive spectral data. Each acquisition cycle consisted of one full MS1 scan followed by 40 sequential MS2 scans. The MS1 scan range was 350–1550 *m*/*z* with a resolution of 120,000. The normalized automatic gain control (AGC) target value was set at 300%, and the maximum injection time was 100 ms. Variable isolation windows were applied for DIA scans. High-energy collisional dissociation (HCD) was performed with a normalized collision energy of 30%. MS/MS spectra were acquired in automatic scan range mode at a resolution of 30,000, with the AGC target set to 3000% and the maximum injection time set to automatic mode.

The separation of phosphopeptide was carried out using an 80-min gradient. All MS spectra were acquired in data-dependent acquisition (DDA) mode, a method that selectively fragments the most intense peptide ions from each MS1 scan to generate MS2 spectra, with the acquisition cycle consisting of one full MS1 scan followed by 40 MS2 scan events. For the MS1 scan, the scan range was 350–1550 *m*/*z* at a resolution of 120,000, with a normalized AGC target of 100%, The maximum injection time was 300 ms and the dynamic exclusion duration was 30 s. For the MS2 scan, precursors with charge states of 2–6 were fragmented by the HCD with normalized collision energies of 21%, 27%, 33%. The first mass of the MS/MS scan was set at 110 *m*/*z* with a resolution of 30,000. The normalized AGC target was set at 100%, and the maximum injection time was set at 50 ms.

### 4.9. MS Data Analysis

MS data acquired in DIA mode were processed using Spectronaut 16.2 (Biognosys, Schlieren, Switzerland), searching against the Rattus norvegicus UniProt database (UP000002494, 22,367 entries, downloaded 16 December 2024) with default search parameters. The false discovery rates (FDRs) at the protein and peptide level were set to 1%.

DDA data were analyzed via MaxQuant 1.6.14 using the same UniProt database. Variable modifications included oxidation (M), N-terminal acetylation, phosphorylation (S/T/Y), and deamidation (N); fixed modification: carbamidomethylation (C); enzyme: Trypsin/P. Match-between-runs and label-free quantification were enabled, with other parameters at defaults. The FDRs at the protein and peptide level were set to 1%. A phosphorylated site localization probability greater than 0.75 were considered as high-confident identifications.

Differential expression analysis of proteins/phosphosites was performed using the Perseus 1.6.10.0. Data preprocessing included filtering out reverse sequences and contaminants, Log_2_ transformation, and imputation of missing values by drawing random numbers from a normal distribution. A Student’s *t*-test (*p* < 0.05) was employed in combination with the Benjamini-Hochberg correction method to control for multiple testing. The specific procedure involved ranking the raw *p*-values from the t-tests of all proteins/phosphosites in ascending order, calculating critical values, and designating items satisfying *p*-value ≤ critical value as statistically significant, ultimately controlling the FDR at <0.05. A threshold of |Log_2_FC| > 1 was simultaneously applied, collectively serving as the criteria for determining statistical significance.

Differentially expressed proteins underwent functional enrichment analysis using Metascape [51] with GO terms and KEGG pathway annotation.

## 5. Conclusions

Through establishing a lethal insulin overdose rat model and integrating spatial proteomics, global proteomics, and phosphoproteomics analyses, this study systematically elucidated the multi-organ molecular response network triggered by insulin overdose. Spatially resolved proteomics precisely identified significant downregulation of digestive zymogens in pancreatic acini and aberrant expression of key transcription factors in islets at the microstructural level, providing spatial evidence for pathological alterations. Global proteomics further validated these findings at the whole-tissue level and revealed robust acute-phase responses and metabolic dysregulation in the liver, indicating distinct organ-specific adaptations to insulin overdose. Phosphoproteomics uncovered potential molecular regulatory mechanisms from a signal transduction perspective, demonstrating significant remodeling of insulin signaling pathways and phosphorylation modifications in other key pathways in both the pancreas and liver. These three layers form a multidimensional evidence chain that provides systematic insights into the molecular mechanisms of insulin overdose-induced hepatic and pancreatic injury.

## Data Availability

The mass spectrometry proteomics data have been deposited to the ProteomeXchange Consortium (https://proteomecentral.proteomexchange.org, accessed on 8 September 2025) via the iProX partner repository [52,53] (http://www.iprox.org, IPX0013276000, accessed on 8 September 2025) with the dataset identifier PXD068162.

## Supplementary Materials

The following supporting information can be downloaded at: https://www.mdpi.com/article/10.3390/ijms262211018/s1.

## Abbreviations

The following abbreviations are used in this manuscript:

ACN	Acetonitrile
AGC	Automatic gain control
Amy2	Alpha-amylase
AP	Acute pancreatitis
BCA	Bicinchoninic acid
BP	Biological process
CaCl_2_	Calcium chloride
CRP	C-reactive protein
DDA	Data-dependent acquisition
DIA	Data-independent acquisition
DTT	Dithiothreitol
FA	Formic acid
FDRs	False discovery rates
FFPE	Formalin-fixed paraffin-embedded
GO	Gene ontology
HCD	High-energy collisional dissociation
HE	Hematoxylin and Eosin
IAA	Iodoacetamide
IACUC	Institutional Animal Care and Use Committee
LCM	laser microdissection
LC-MS/MS	Liquid chromatography–tandem mass spectrometry
KEGG	Kyoto Encyclopedia of Genes and Genomes
NaCl	Sodium chloride
NaF	Sodium fluoride
Na_3_VO_4_	Sodium orthovanadate
NH_4_OH	Ammonium hydroxide
PBS	Phosphate-buffered saline
PCA	Principal component analysis
PCR	Polymerase chain reaction
PFA	Paraformaldehyde
PPI	Protein-protein interaction
Prss1	Serine protease 1
Prss2	Anionic trypsin-2
RT	Room temperature
SD	Sprague-Dawley
SISPROT	Simple and Integrated Spintip-based Proteomics Technology
T1DM	Type 1 diabetes mellitus
Ti^4+^-IMAC	Ti^4+^-immobilized metal affinity chromatography
TFA	Trifluoroacetic acid
