# Supplementary material for: Spatial- and Phospho-Proteomic Profiling Reveals Pancreatic and Hepatic Dysfunction in a Rat Model of Lethal Insulin Overdose
Source: Int J Mol Sci. 2025 Nov 14;26(22):11018. doi: 10.3390/ijms262211018 (PMC12652034; doi:10.3390/ijms262211018)
Supplement: Supplementary file 1 [file ijms-26-11018-s001.zip › ijms-3977762-supplementary.pdf]

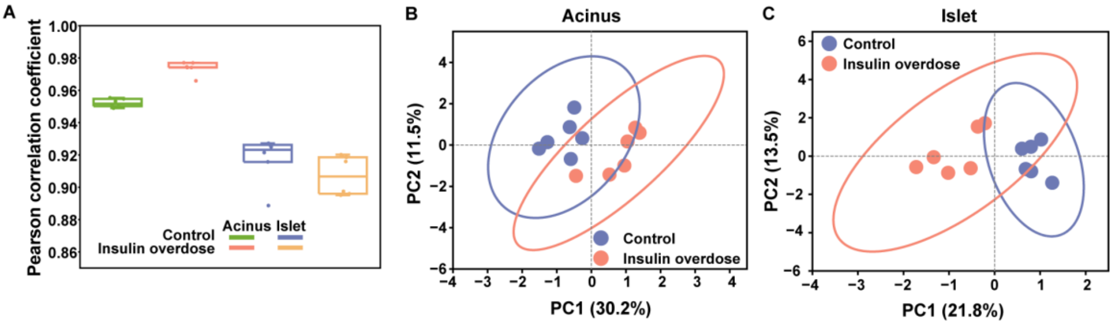

Figure S1. Excellent intra-group reproducibility and inter-group separation between control and insulin-overdose groups in acinar and islet. **A**: Pearson correlation coefficient analysis shows high intra-group consistency in the data obtained from both acinar and islet tissues across all experimental groups, demonstrating stable experimental operations and reliable data; n = 6 per group. **B**-**C**: Principal component analysis reveals that insulin overdose induces global alterations in the protein expression profiles in both (**B**) acinar and (**C**) islet, manifested as clear separation between the insulin overdose and control groups; n = 6 per group.

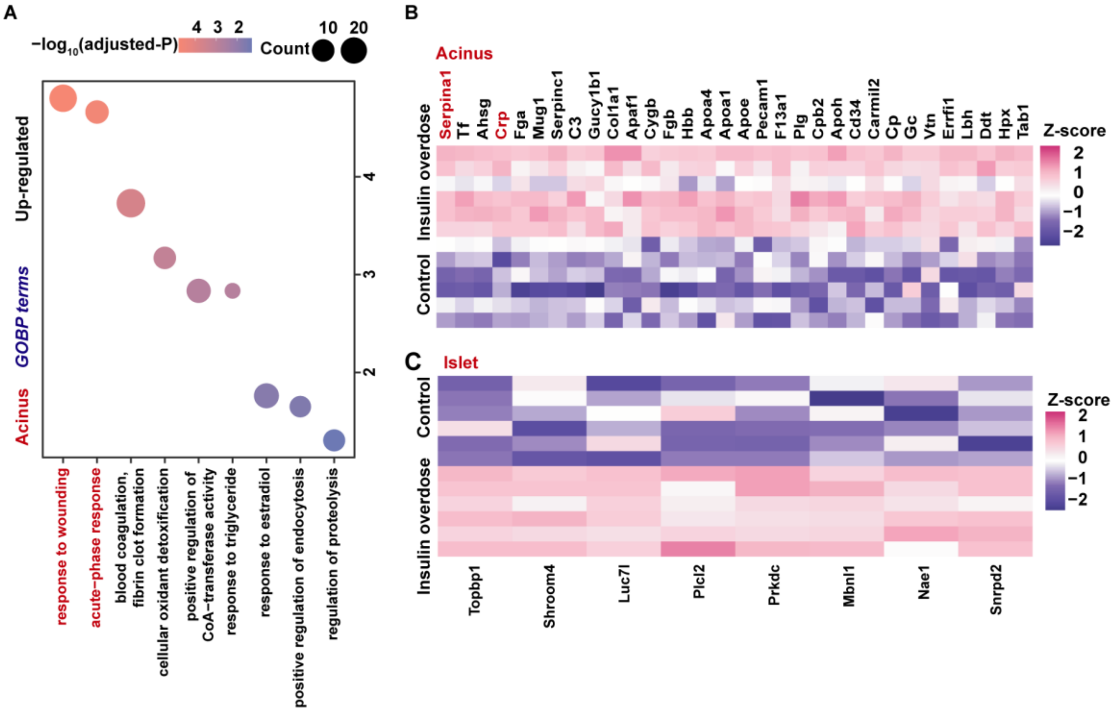

Figure S2. Activation of acute-phase response pathways in acinar and upregulation of specific proteins in islets induced by insulin overdose. **A**: Bubble plot of GOBP enrichment analysis for upregulated DEPs in acinar tissue. Dot size corresponds to the number of proteins enriched per term, and color represents the -log10(adjusted P). Results show significant enrichment of upregulated proteins in acute injury-related pathways such as "response to wounding" and

"acute-phase response". **B**: Heatmap showing upregulated proteins associated with the "response to wounding" and "acute-phase response" pathways in acinar tissue. The heatmap clearly demonstrates coordinated upregulation of acute-phase response proteins, including Serpina1 and Crp, in the insulin-overdose group. Protein expression levels were Z-score. **C**: Heatmap displaying all upregulated DEPs in islet tissue. Only a small number of proteins were specifically upregulated in the islets. Protein expression levels were Z-score.

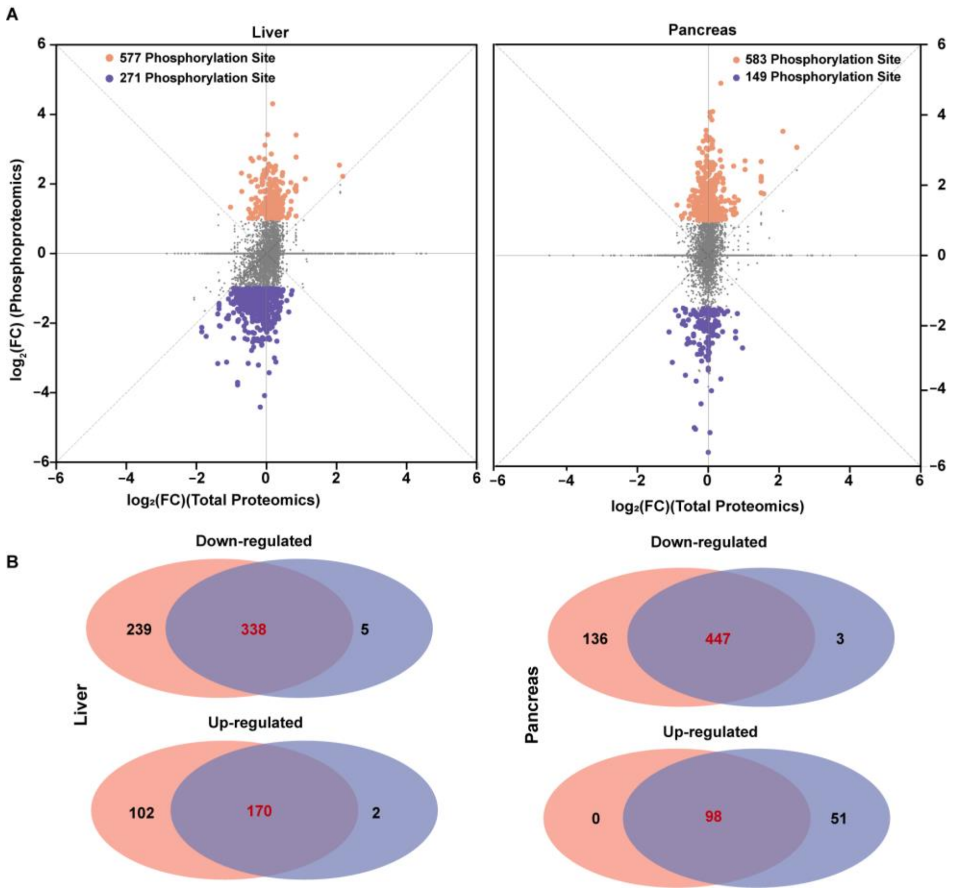

Figure S3. Identification of specifically phosphorylated sites in the insulin-overdose group within the liver and pancreas. **A**: Scatter plots depicting the association between the magnitude of change for each phosphorylation site [log_2FC(Phosphorylation)] and the magnitude of change for its corresponding total protein [log_2FC(Total protein)] in the insulin overdose group (Left: liver; Right: pancreas). Orange and purple data points represent phosphorylation sites that simultaneously satisfy the following two criteria: (1) significant change in the differential analysis between the insulin-overdose and control groups [log_2FC(phosphorylation) > 1 or < -1]; and (2) the magnitude of its change is greater than that

of the corresponding total protein [|$\log_2$FC(phosphorylation)| > |$\log_2$FC(total protein)|]. This analysis serves as a preliminary screening step aimed at identifying candidate phosphorylation sites potentially governed by post-translational modifications based on the magnitude of change. The statistical significance of these candidate sites requires final confirmation through subsequent intersection with results meeting the FDR < 0.05 criterion. **B**: Venn diagram displaying the overlap between proteins filtered from Chart A and proteins meeting the screening criteria of phosphorylation data (FDR < 0.05 and $\log_2$FC > |1|) (Downregulation: Upper quadrant; Upregulation: Lower quadrant). This intersection represents the final screened set of phosphorylation sites that are not only statistically significant but also exhibit changes in phosphorylation levels independent of alterations in their corresponding total protein expression.

Table S1 Quantification of laser microdissected acinar and islet areas in the control and insulin-overdose groups.

Islet	Area (μm^2)	Islet	Area (μm^2)	Acinus	Area (μm^2)	Acinus	Area (μm^2)
C_1	500,432	I_1	501,917	C_1	500,308	I_1	502,233
C_2	500,386	I_2	500,802	C_2	502,890	I_2	502,562
C_3	500,304	I_3	500,030	C_3	502,532	I_3	505,215
C_4	502,426	I_4	501,077	C_4	503,206	I_4	501,452
C_5	503,222	I_5	500,190	C_5	498,355	I_5	498,527
C_6	500,205	I_6	500,872	C_6	504,348	I_6	505,513

C: Control; I: Insulin overdose

This table presents the area measurements (μm^2) of pancreatic regions (islet and acinus) isolated by laser microdissection across experimental groups. The absence of significant differences in area measurements both within and between groups demonstrates the reproducibility and comparability of the sampling operation, ensuring the reliability of subsequent spatial proteomic analysis.
